# YOLOV5-CBAM-C3TR: an optimized model based on transformer module and attention mechanism for apple leaf disease detection

**DOI:** 10.3389/fpls.2023.1323301

**Published:** 2024-01-15

**Authors:** Meng Lv, Wen-Hao Su

**Affiliations:** College of Engineering, China Agricultural University, Beijing, China

**Keywords:** deep learning, apple leaf, disease detection, YOLOv5, attention mechanism, transformer encoder

## Abstract

Apple trees face various challenges during cultivation. Apple leaves, as the key part of the apple tree for photosynthesis, occupy most of the area of the tree. Diseases of the leaves can hinder the healthy growth of trees and cause huge economic losses to fruit growers. The prerequisite for precise control of apple leaf diseases is the timely and accurate detection of different diseases on apple leaves. Traditional methods relying on manual detection have problems such as limited accuracy and slow speed. In this study, both the attention mechanism and the module containing the transformer encoder were innovatively introduced into YOLOV5, resulting in YOLOV5-CBAM-C3TR for apple leaf disease detection. The datasets used in this experiment were uniformly RGB images. To better evaluate the effectiveness of YOLOV5-CBAM-C3TR, the model was compared with different target detection models such as SSD, YOLOV3, YOLOV4, and YOLOV5. The results showed that YOLOV5-CBAM-C3TR achieved mAP@0.5, precision, and recall of 73.4%, 70.9%, and 69.5% for three apple leaf diseases including Alternaria blotch, Grey spot, and Rust. Compared with the original model YOLOV5, the mAP 0.5increased by 8.25% with a small change in the number of parameters. In addition, YOLOV5-CBAM-C3TR can achieve an average accuracy of 92.4% in detecting 208 randomly selected apple leaf disease samples. Notably, YOLOV5-CBAM-C3TR achieved 93.1% and 89.6% accuracy in detecting two very similar diseases including Alternaria Blotch and Grey Spot, respectively. The YOLOV5-CBAM-C3TR model proposed in this paper has been applied to the detection of apple leaf diseases for the first time, and also showed strong recognition ability in identifying similar diseases, which is expected to promote the further development of disease detection technology.

## 1 Introduction

Apples are highly prized for their nutritional richness and rank among the world’s most economically significant fruits (Shu et al., 2019). However, due to environmental, bacterial, and insect pests, the growth of apple fruits can be attacked by a variety of diseases, which can lead to a decrease in fruit yield and quality, resulting in huge economic losses. Timely detection and accurate classification of the type of disease is the first step to early control of the disease. The leaves of apple trees occupy most of the area of the tree and are the easiest part to observe. Most apple diseases can be identified by observing diseased leaves (Wang et al., 2009; Vishnu and Rajanith, 2015). Therefore, the research in this study focuses on diseases of apple leaf parts.

Traditionally, the identification of apple leaf disease mostly relied on experienced farmers to identify the disease. However, due to the similarity of diseases or the complexity of symptoms, relying on human eye detection can easily lead to misjudgment of diseases, which can not only solve the problem of diseases but also cause environmental pollution (Liu et al., 2022). The combination of machine learning and image processing replaced human eye detection and provided a new direction for disease detection. For example, Dubey and Jalal (2012) used K-means clustering for the segmentation of apple fruit diseases, then global color histogram, color coherence vector, local binary pattern, and complete local binary pattern were used for feature extraction, the support vector machine (SVM) (Hearst et al., 1998) was used for disease classification, which can achieve an accuracy of 93%. Chuanlei et al. (2017) introduced a method for apple leaf disease detection. To improve the detection accuracy, a region-growing algorithm is used to segment the disease image, a genetic algorithm combined with correlation feature selection is used to select the important features, and finally SVM classifier is used to identify the disease, which was tested on a dataset containing 90 images on a dataset with an accuracy of 90%. Shi et al. (2017) proposed an apple disease recognition method based on two-dimensional subspace learning dimensionality reduction, with recognition accuracy above 90% on the apple leaf disease dataset. Gargade and Khandekar (2021) used K-NN and SVM algorithms to classify apple leaf defects with 99.5% accuracy. Jan and Ahmad (2020) used 11 apple leaf image features and a multilayer perceptron (MLP) pattern classifier to detect apple Alternaria leaf blotch with 99.1% accuracy. However, segmentation based on image processing and feature extraction based on traditional machine learning are extremely complex, leading to inefficient disease diagnosis.

In recent years, convolutional neural network (CNN)-based model avoids complex preprocessing work on images and automatically extracts features through an end-to-end approach (Abade et al., 2021; Dhaka et al., 2021), which is more suitable for solving problems in the field of computer vision. Apple leaf disease detection tasks can be classified into three main categories according to the type of computer vision tasks: 1) image classification, which classifies the detected images into various types of diseases, 2) target detection, which detects and locates the diseases in the images, and 3) image segmentation, which segments the images into semantic disease maps. In general, image classification studies using CNN models are the most common. Based on Densenet-121, Zhong and Zhao (2020) proposed regression, multi-label classification and focal loss function recognition methods for three apple leaf diseases with accuracies of 93.51%, 93.31% and 93.71%, respectively. Yu and Son (2019) used the ROI-aware DCNN model to classify Marssonia blotch and Alternaria leaf spots, which was shown to outperform traditional methods. Singh et al. (2021) improved the classical CNN model to implement Marssonia Coronaria, Rust, and Scab for accurate classification with up to the accuracy of 99.2%. Babu and Ram (2022) proposed a deep residual convolutional neural network (DRCNN) with contrast limited adaptive histogram equalization for weed and soybean crop classification with an accuracy of 97.25%. Kundu et al. (2021) proposed the use of deep learning in conjunction with IoT for automatic detection of pearl millet diseases, and the accuracy of the proposed custom network model is comparable to that of the current state-of-the-art image classification model, with an accuracy of up to 98.78%, while greatly reducing the training time. Image classification methods are excellent at accurately classifying diseases, but their utility is limited by failing to provide information about the location of the disease. In contrast, target detection methods can locate the target object in real-time and obtain more detailed information, which is more conducive to practical application. Currently, target detection methods can be classified into single-stage and two-stage algorithms. Two-stage algorithms such as Faster-RCNN (Ren et al., 2015) and Mask-RCNN (Kaiming et al., 2017) have higher detection accuracy but lose detection speed. In comparison, single-stage algorithms are characterized by a small number of model parameters and fast inference speed, which better meet the needs of practical production environments. The single-stage algorithms are best known as you only look once (YOLO) (Redmon et al., 2016), which turns the detection task into a simple regression problem and has a simple network model that is easy for researchers to learn and train. Although there have been many iterations of YOLO, YOLOV5 remains the most widely used version across all domains (Lang et al., 2022). For example, Chen et al. (2022) added the SE module to YOLOV5 and replaced the original loss function GIOU with EIOU to automatically identify diseases on rubber trees, finally the average accuracy was improved by 5.4% compared to the original YOLOV5. With the aim of improving the accuracy of vegetable disease detection in natural environments, Li (2022) improved the CSP, FPN, and NMS modules in YOLOV5s, and finally achieved a mAP of up to 93.1% on a dataset containing a total of 1,000 images of five diseases. In order to accurately identify and locate tomatoes, Li et al. (2023) optimized YOLOV5 by adding the CARAFE module to obtain a larger sensory field while maintaining lightness, introducing EIOU and quality focal loss to solve the problem of uneven samples, and finally proposing YOLOv5s-CQE. The mAP 0.5of YOLOv5s-CQE on the dataset containing 3,820 tomato images finally reaches 98.68%. Therefore, YOLOV5 shows excellent detection accuracy and fast processing speed in a series of target detection tasks and shows great potential in the automatic identification and classification of apple leaf diseases.

In this study, aiming to achieve accurate detection of three common apple leaf diseases in the natural environment, YOLOV5 was selected as the baseline model, and YOLOV5-CBAM-C3TR was finally proposed by adding different attention mechanisms and C3TR modules and transformer encoders individually or jointly. The specific objectives of this study are as follows: (1) A proposal for an improved YOLOV5 method based on CBAM and C3TR modules for the identification of three apple leaf diseases including Alternaria blotch, Grey spot, and Rust; (2) Comparison of the performance of YOLOV5-CBAM-C3TR, SSD, YOLOV3, YOLOV4, YOLOV5 and other different target detection models on the same dataset containing three diseases; (3) Comparison of the performance improvement of YOLOV5 with the addition of CBAM, SE, ECA and C3TR modules, individually or in combination; (4) development of a model for the effective classification of similar apple leaf diseases. As far as we know, this is the first time that the YOLOV5-CBAM-C3TR model has been used for the identification and localization of apple leaf diseases.

## 2 Materials and methods

### 2.1 Datasets

In this study, the images were collected from the publicly available apple leaf pathology image dataset (https://aistudio.baidu.com/datasetdetail/11591). Disease images in natural environments in the dataset were obtained from a real apple orchard in Yantai, Shandong Province, China. A total of 390 high-quality images of three common apple leaf diseases were selected for study in this dataset. However, the original images cannot be trained, validated, and tested directly. Images used for target detection need to determine the location of the target in the dataset image, which requires the researcher to label the observed targets before starting training, validation, and testing (Wang and Zhao, 2022). The dataset used in this study was in YOLO format and manually labeled for apple leaf diseases using image annotation software. In order to facilitate model training, the labeled images were divided into training, validation, and test sets in a ratio of 8:1:1. In addition, to better adapt the model to different environments and to reduce the negative effects of photometric distortion during training (Zhu et al., 2021), data enhancements such as random cropping, panning, changing luminance, adding noise, rotating, and mirroring were chosen to extend the dataset. Finally, the dataset required for the experiment consisted of 3900 images of apple leaves containing the three diseases, 1680 from the laboratory background and 2220 from the orchard background, as shown in **Table 1**.

**Table 1:** Apple leaf disease dataset.

Diseases	Training Set	Validation Set	Test Set	Total
Alternaria blotch	1040	130	130	1300
Grey spot	1040	130	130	1300
Rust	1040	130	130	1300
Total	3120	390	390	3900

### 2.2 Methods

#### 2.2.1 YOLOV5

**Figure 1:**
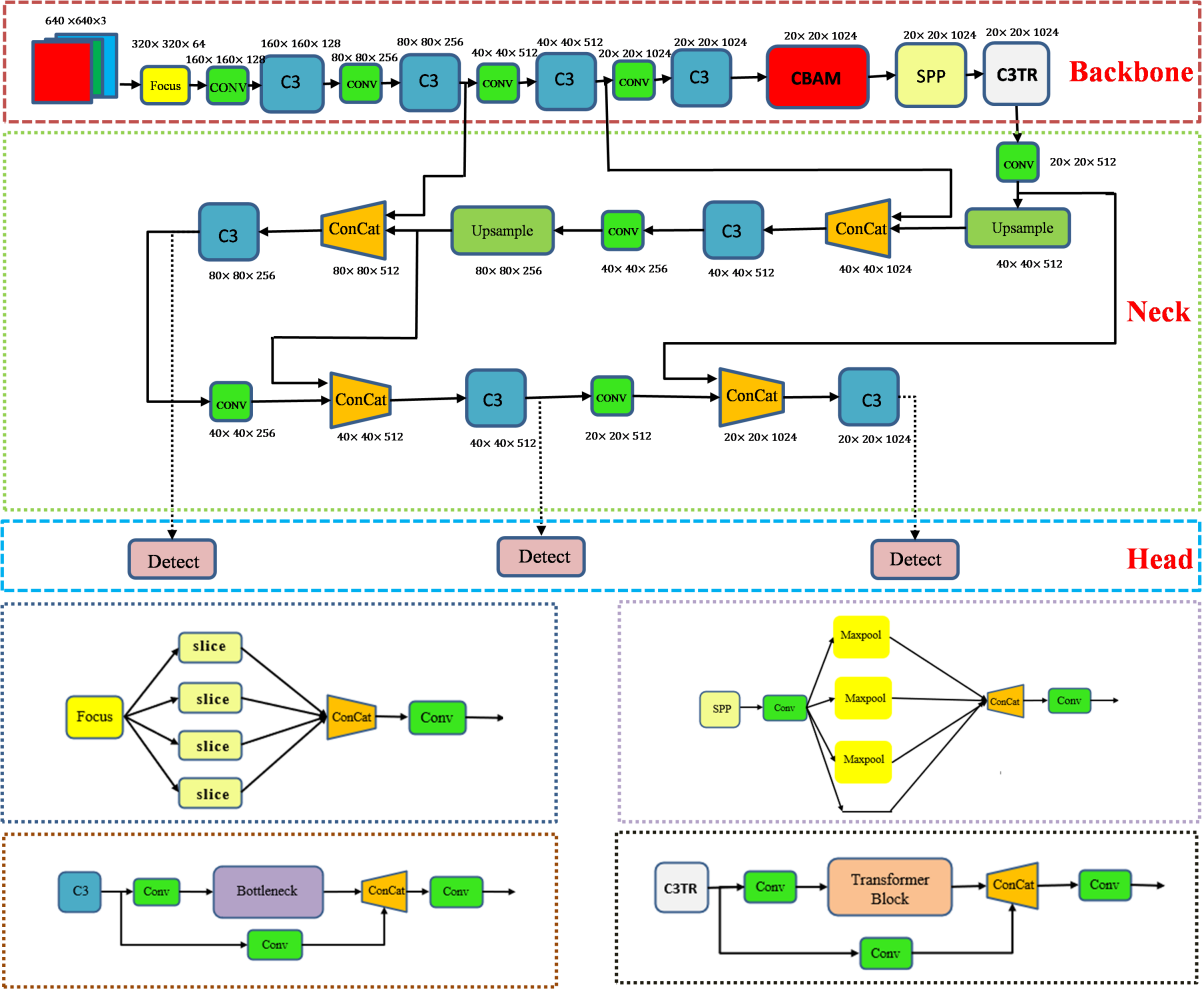
Overall structure of YOLOV5-CBAM-C3TR.

The aim of this study is to achieve real-time detection and accurate identification of apple leaf diseases. Considering the type of disease detected including early-stage disease, the shape of the infestation is small. Therefore, target detection methods are chosen for identification. Classical single-stage target detection algorithms such as SSD (Liu et al., 2016), YOLOV3 (Redmon and Farhadi, 2018), YOLOV4 (Bochkovskiy et al., 2020), YOLOV5 (Jocher et al., 2021), RetinaNet (Lin et al., 2017) can obtain the positional information of the target object for identification and localization. In this study, YOLOV5 was selected for the detection of apple leaf diseases. As shown in **Figure 1**, the YOLOV5 model consists of four parts: Input, Backbone, Neck, and Head. The main work of each part is as follows:

1. Input. The Input part of YOLOV5s is preprocessed by adding mosaic data enhancement, adaptive anchor frames, and adaptive image scaling. The model can extract the features better during training and thus shows better results on the dataset.
2. Backbone. The Backbone part mainly relies on the Focus, C3, and SPP modules to extract features from the input images. The Focus module performs slicing operations on the image before it enters the backbone, thus reducing the feature dimensionality. The C3 module, which consists of three convolutional modules and a bottleneck structure, brings the dual advantages of increased computational speed and reduced parameter complexity. The SPP module is a pooling module that passes the input features in parallel to the Maxpool pooling layer to obtain a set of feature maps of different sizes, and finally joins these feature maps together so that feature information at different scales can be captured. The backbone is responsible for passing the extracted position and category information to the Neck layer.
3. Neck. The Neck part of the YOLOV5s combines up-sampling and down-sampling to generate a feature pyramid that improves the detection accuracy of the target object, which on one hand needs to reprocess the extracted features in the backbone network and on the other hand plays an important role in the subsequent detection.
4. Head. The Head part is to classify and predict the results of the neck layer by using a 1 × 1 convolutional layer to generate batch size different three results for final target detection.

#### 2.2.2 CBAM module

**Figure 2:**
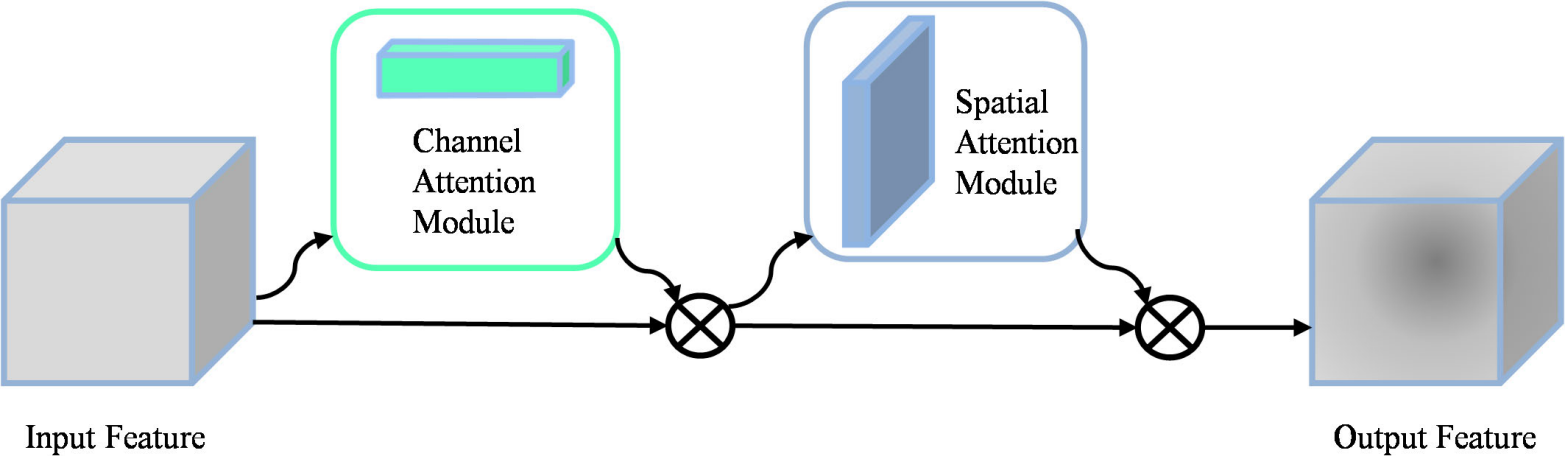
Specific structure of the CBAM module.


When detecting apple leaf diseases, intricate background environments can cause interference, which can affect the accuracy of disease recognition. To address this challenge, integrating the attention mechanism becomes a promising solution that enhances the model’s ability to selectively focus on relevant features while filtering out irrelevant information. As shown in **Figure 2**, the convolutional block attention mechanism (CBAM) (Woo et al., 2018) consists of two key components: the channel attention module (CAM) and the spatial attention module (SAM). The CAM emphasizes the key features, while the SAM emphasizes the spatial localization of these key features. The operation of the CAM consists of extracting features through average pooling and maximum pooling respectively. These features are then processed separately through a MLP network, and finally summed and output the feature vector. The mathematical formulation of the Channel Attention Module was shown in Equation 1.

(1) $$M_{c}\left(F\right)=\sigma \left(\text{MLP}\left(\text{AvgPool}\left(F\right)\right)+\text{MLP}\left(\text{MaxPool}\left(F\right)\right)\right)$$

where σ is a nonlinear sigmoid function used to map inputs to continuous outputs between 0 and 1. F is the input feature map, the MLP consists of two linear layers and a ReLU activation function.

SAM generates the spatial attention map by splicing the features that are average pooled and maximum pooled in the channel dimension. The formulation of the Spatial Attention Module was shown in Equation 2.


(2) $$M\text{s}\left(F\right)=\sigma \left(f^{7\times 7}\left(\text{Concat}\left(AvɡPool\left(F\right),Maxpool\left(F\right)\right)\right)\right)$$

Where σ denotes the sigmoid function, $f^{7\times 7}$ represents a convolutional kernel size of 7 × 7, and Concat denotes the connection operation.

#### 2.2.3 C3TR module

In recent years, the transformer (Vaswani et al., 2017) architecture has been widely used in the field of natural language processing (NLP) with resounding success. As with NLP, where large amounts of textual data are key to training, the field of computer vision also relies on large image libraries for effective model learning. The transformer module can acquire complex relationships between different locations in the image. The multiple attention mechanism in the transformer module helps to extract multi-scale information, which can focus on both location and feature information and has great research potential. In order to realize the application of transformer in the field of computer vision, researchers endeavor to replace certain convolutional structures with transformer. For example, in target detection involving a drone capture scene, Zhu et al. (2021) innovatively integrated the transformer block into the C3 module of the YOLOV5 architecture, resulting in the C3TR module. As shown in **Figure 1**, richer image information extraction is achieved by replacing the bottleneck module in the C3 module.

**Figure 3:**
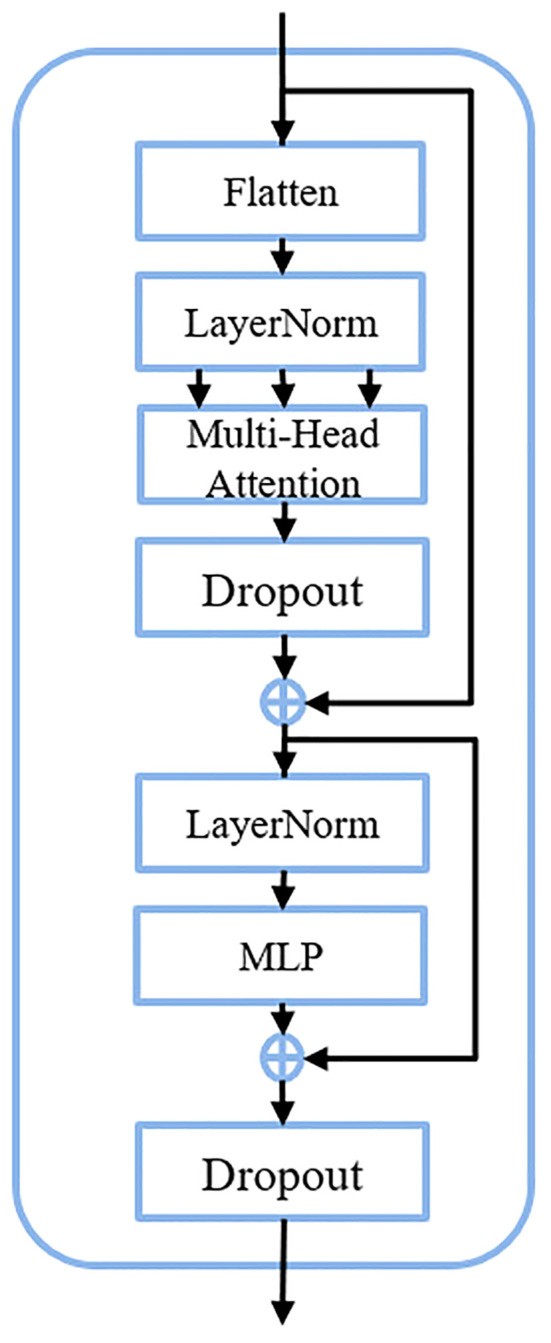
Detailed architecture of the transformer block.

The transformer block serves as the fundamental constituent within the C3TR framework, adopting the classical transformer encoder architecture. Illustrated in **Figure 3**, this block is comprised of three primary layers: Flatten, Multi-head attention, and feedforward neural network (FFN).

1. Flatten

The Flatten operation is to flatten the two-dimensional feature vectors obtained by the model based on the image into one-dimensional vectors, which can preserve the positional information of the image. If an input feature map $X\in R^{H\times W\times C}$ is given, it will become $X_{1}\in R^{H\times C}$ after the spreading operation, where H×W=H.

2. Multi-head attention

The multiple attention operation is responsible for different linear mappings through the Flatten and LayerNorm, allowing simultaneous attention to feature information at different scales. After converting the feature maps into $Q,K,V\in R^{N\times C}$ as inputs for multi-head attention, each single head performs one feature mapping for Q, k, V. The output formula after the completion of the single-head attention operation was shown in Equation 3 and Equation 4.


(3) $$Output_{i}=S_{i}V_{i}$$

(4) $$S_{i}=\text{softmax}\left(Q_{i}K_{i}^{T}\right)$$

where $Q_{i},K_{i},V_{i}$ denote the multiplication of Q, K, V with the weight matrix of the single-head attention mechanism, $S_{i}$ represents the single-head attention matrix, and $Output_{i}$ refers to the integration of global information. The $Output_{i}$ generated after feature mapping for each single-head attention will eventually be unified through the connectivity layer to produce the final output with expression was shown in Equation 5.


(5) $$Output=\text{Conca t}\left(Output_{1},Output_{2},\mathrm{...},Output_{n}\right)$$

where n represents the number of multi-head attention.

3. FFN

The FFN layer is a feed forward neural network, which is composed of two fully connected layers, one of which contains the Relu activation function and the Dropout function between the two layers. The expression of FFN was shown in Equation 6.


(6) $$FFN\left(x\right)=max\left(0,xW_{1}+b_{1}\right)W_{2}+b_{2}$$

where x represents the feature sequence of the input FFN layer, $W_{1}$ and $b_{1}$ represent the weights and offsets of the first fully connected layer, and $W_{2}$ and $b_{2}$ represent the weights and offsets of the second fully connected layer, respectively.

#### 2.2.4 Proposed model

Orchard environments are extremely complex. Common problems in target object detection such as similar texture between target object and background, target occlusion, and similarity between target object types. The focus on improving detection accuracy led us to optimize the YOLOV5 framework. This was done by trying to add CBAM, SE (Hu et al., 2018), ECA (Wang et al., 2020), and C3TR modules to improve the performance of the model. Finally, by adding CBAM module before SPP module and C3TR module at the last layer of backbone network, the optimized YOLOV5-CBAM-C3TR model was proposed. **Figure 1** shows the overall structure of the optimized model YOLOV5-CBAM-C3TR. Before starting the training, the optimal runtime environment was created, the input images were resized to 640 × 640. After training, the final three different dimensions of the detection header effectively outputted important information related to the type and location of the apple leaf disease.

### 2.3 Model training environment parameter configuration

In this study, the model training environment was built using Pytorch and GPUs with the parameters shown in **Table 2**. The adaptive moment estimation (Adam) (Kingma and Ba, 2014) was used as the optimizer in the experiments. The input image input size was set to 640×640, obtained by filling the original image. After repeated experiments, the final hyperparameters were set as follows: the initial learning rate was set to 0.0005, the epoch number was set to 100, and the batch size was 8. To ensure the fairness of model comparison, the parameters used in this study were consistent.

**Table 2:** Software and hardware environment resource configuration.

Configuration	Parameter
Operating system	Ubuntu 20.04
GPU	NVIDIA GeForce RTX 3090
CPU	Intel(R) Xeon(R) Gold 6330 CPU @ 2.00GHz
Memory	100 GB
Language	Python 3.8
Framework	Pytorch 1.10.0

### 2.4 Model evaluation

In order to comprehensively assess the performance of the model in apple leaf disease detection, a set of evaluation metrics including precision, recall, mAP@0.5, mAP@[0.5:0.95], F1 Score, and parameters were chosen. Among them, mAP is the mean average precision, which is the evaluation metric of the main model in target detection. mAP 0.5and mAP@[0.5:0.95] are distinguished by the difference in the size of the intersection over union (IOU), which determines that mAP@[0.5:0.95] is more stringent. In addition, the loss value is used to assess the error between the predicted and the ground truth. The training loss reflects the model’s ability to fit on that dataset, and the validation loss reflects the model’s ability to generalize. The loss value contains three parameters: obj_loss (object loss), cls_loss (classification loss), box_loss (bounding loss). The above metrics were calculated in Equation 7, Equation 8, Equation 9, Equation 10, and Equation 11.


(7) $$Precision=\frac{TP}{TP+FP}$$

(8) $$Recall=\frac{TP}{TP+FN}$$

(9) $$F1\;Score=\frac{2\times Precision\times Recall}{Precision+Recall}$$

(10) $$mAP=\frac{\sum_{i=1}^{C}AP_{i}}{C}$$

(11) $$loss=box_{\_}loss+obj\_loss+cls\_loss$$

where TP , FP , FN , and TN stand for true positive, false positive, false negative, and true negative, respectively. The $AP_{i}\;$ is the average precision value at the i-th species. C is the total number of species.

## 3 Results

### 3.1 Model optimization

To further improve the detection precision of YOLOV5 for apple leaf diseases, different modules including SE, CBAM, ECA, and C3TR were added to improve the detection capability of YOLOV5. As can be seen from **Table 3**, compared with the original YOLOV5 model, the improved YOLOV5-CBAM-C3TR achieved 73.4%, 40.9%, 70.9%, and 69.5% of mAP@0.5, mAP@[0.5:0.95], precision and recall, which was a significant improvement in detection performance. In addition, the experimental results also showed that the improved YOLOV5-CBAM-C3TR is more suitable for the detection of apple leaf diseases in real and complex environments.

**Table 3:** Comparison with different target detection models.

Methods	mAP@0.5 (%)	mAP@ [0.5:0.95] (%)	Precision (%)	Recall (%)
SSD	66.2	**46.9**	**96.8**	36.9
YOLOV3	65.1	33.3	64.3	63.4
YOLOV4	52.4	18.6	87.9	25.1
YOLOV5	67.8	34.9	67.3	66.1
MGA-YOLOV5	69.0	34.2	74.4	64.3
BTC-YOLOV5	72.0	39.8	70.7	67.9
**YOLOV5-CBAM-C3TR**	**73.4**	40.9	70.9	**69.5**

The performance of the indicators is best shown in bold.

#### 3.1.1 Model performance optimization by adding an individual module

As can be seen from **Table 4**, the low accuracy of YOLOV5 in detecting apple leaf diseases may be caused by the fact that the two diseases including Alternaria blotch and Grey spot, which were too similar. Therefore, to make the model more focused on extracting the characteristics of apple leaf disease, different attention mechanisms were tried to be added to the backbone network of YOLOV5 for experiments. As shown in **Table 4**, the addition of SE, ECA, CBAM and C3TR all improved the accuracy of the YOLOV5 model for detecting apple leaf diseases while keeping the number of model parameters relatively constant. The SE module allows the model to better focus on feature channels that are effective for apple leaf disease identification. Compared to YOLOV5, the addition of the SE model resulted in an improvement of 3.39%, 5.73%, -4.75%, -5.3%, and -5.1% in mAP@0.5, mAP@[0.5:0.95], precision, recall, and F1 score, respectively. The ECA module calculates the correlation of the feature channels so that the model focuses more on the desired feature channels. Compared to YOLOV5, the addition of the ECA model resulted in an improvement of 3.69%, 6.88%, 5.65%, -2.12%, and 1.5% in mAP@0.5, mAP@[0.5:0.95], precision, recall, and F1 score, respectively. Unlike the SE and ECA modules, The CBAM module extracts features by focusing on the channel and spatial information of the image. Compared to YOLOV5, the addition of the CBAM model resulted in an improvement of 7.08%, 12.03%, -7.43%, -7.72%, and -7.65% in mAP@0.5, mAP@[0.5:0.95], precision, recall, and F1 score, respectively. The transformer module in the C3TR module captures global contextual information, which improved the mAP@0.5, mAP@[0.5:0.95], precision, recall, and F1 score of the C3TR module by 6.34%, 15.2%, 8.9%, 2.7%, and 5.7% over the YOLOV5 model, respectively. Adding modules can improve the accuracy of the model in detecting target objects, but it also increases the number of parameters of the model, which is not conducive to the lightweight deployment of the model. By observing the change in the number of model parameters when each module acts alone. It was found that the addition of the SE module severely increased the number of model parameters, while the addition of the other four modules had little effect on the number of model parameters.

**Table 4:** Comparison of model performance improvement by adding a single module.

Methods	mAP@0.5 (%)	mAP@ [0.5:0.95] (%)	Precision (%)	Recall (%)	F1 Score	Parameters
YOLOV5	67.8	34.9	67.3	66.1	66.7	**7018216**
YOLOV5-SE	70.1	36.9	64.1	62.6	63.3	7542504
YOLOV5-ECA	70.3	37.3	71.1	64.7	67.7	7018217
YOLOV5-CBAM	**72.6**	39.1	62.3	61.0	61.6	7051627
YOLOV5-C3TR	72.1	**40.2**	**73.3**	**67.9**	**70.5**	7060072

The performance of the indicators is best shown in bold.

#### 3.1.2 Model performance optimization by adding multiple modules

As can be seen from **Table 5**, adding the modules individually all improved the detection accuracy of the YOLOV5 model. In order to further improve the feature extraction ability of the model, the attention mechanism was combined with the C3TR module. The combination experiments of CBAM+C3TR, ECA+C3TR, and SE+C3TR were conducted respectively. **Table 3** shows that combining two modules improved the detection accuracy of the YOLOV5 model better than adding a single module. Compared with the addition of SE and C3TR alone, the mAP@0.5, mAP@[0.5:0.95], precision, recall, and F1 score of the YOLOV5-SE -C3TR were improved by 3.7% and 0.8%, 10.8% and 1.7%, 6.2% and -7.1%, and 8.6% and 0.1%, 7.4% and -3.5%, respectively. Compared with the addition of the ECA module and C3TR module alone, the mAP@0.5, mAP@[0.5:0.95], precision, recall, and F1 score of YOLOV5-ECA -C3TR were improved by 3.0% and 0.4%, 7.8%, and 0.0%, -1.1% and -4.1%, 5.4% and 0.4%, and 2.2% and -1.8%, respectively. Compared with the addition of the CBAM module and C3TR module alone, the mAP@0.5, mAP@[0.5:0.95], precision, recall, and F1 score of the YOLOV5-CBAM-C3TR were improved by 1.1% and 1.8%, 4.6% and 1.7%, 13.8% and -3.2%, 13.9%, and 2.4%, and 14% and -0.4%, respectively. Overall, combining SE, ECA, and CBAM with C3TR all further improved mAP@0.5, mAP@[0.5:0.95], and recall with little parameter change compared to adding each module individually. Although the addition of multiple modules resulted in a decrease in accuracy and F1 score metrics compared to the addition of the C3TR module alone, YOLOV5-CBAM-C3TR had the smallest decrease and the largest increase, achieving almost positive growth and being the best performing model. The added SE or ECA modules need to capture channel information, while the transformer module in C3TR needs to capture context information. The reason for the accuracy degradation may be the mutual interference between multiple modules leading to inadequate feature extraction. On the other hand, the CBAM module, which focuses on both channel and spatial dimension information, interoperates with the C3TR module to better ensure that sufficient feature information is provided to the model.

**Table 5:** Comparison of model performance improvement by adding combinations of models.

Methods	mAP@0.5 (%)	mAP@ [0.5:0.95] (%)	Precision (%)	Recall (%)	F1 Score	Parameters
YOLOV5-SE -C3TR	72.7	**40.9**	68.1	68.0	68.0	7092840
YOLOV5-ECA -C3TR	72.4	40.2	70.3	68.2	69.2	**7060073**
**YOLOV5-CBAM-C3TR**	**73.4**	**40.9**	**70.9**	**69.5**	**70.2**	7093483

The performance of the indicators is best shown in bold.

### 3.2 Model training

**Figure 4:**
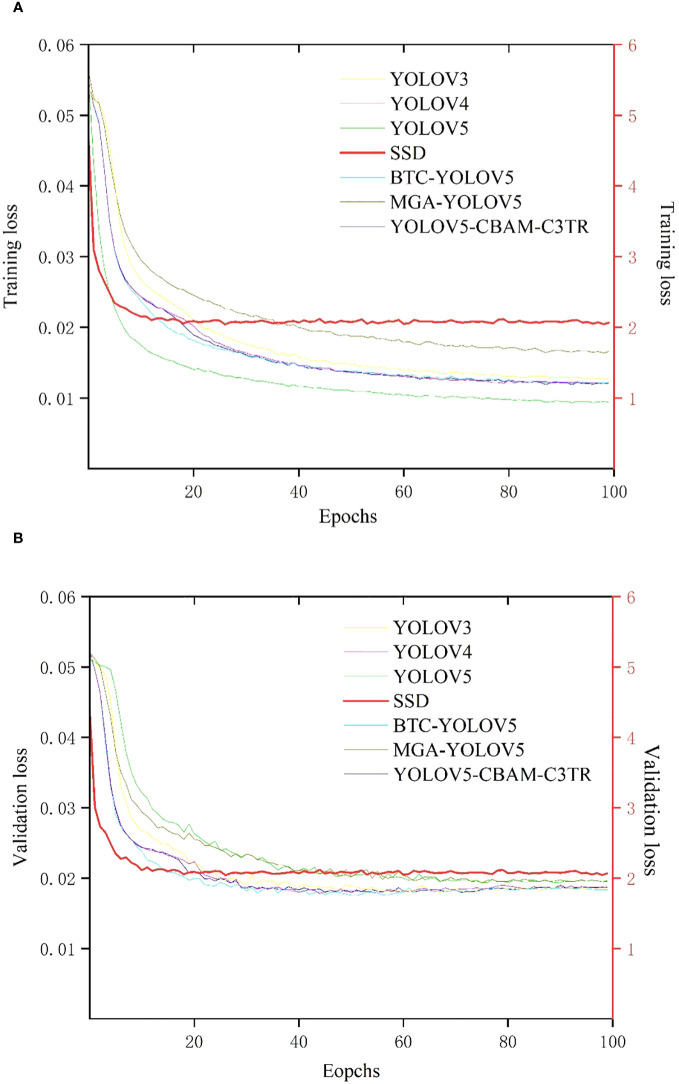
**(A)** Curve of training loss values with epoch values, **(B)** Curve of validation loss values with epoch values. Due to the different scales of change in loss function values for SSD and other models, a double Y-axis is used to represent the change in loss function for each model. The left axis represents the scale of variation of the loss function values for the YOLOV3, YOLOV4, YOLOV5, BTC-YOLOV5, MGA-YOLOV5, and YOLOV5-CBAM-C3TR models, and the right axis represents the scale of variation of the loss function values for the SSD model. the loss function curves for the SSD model are shown in bold red.

Seven target detection models including YOLOV3, YOLOV4, YOLOV5, SSD, MGA-YOLOV5, BTC-YOLOV5, and optimized YOLOV5-CBAM-C3TR were established based on labeled apple leaf disease images. **Figure 4A** shows the graph of training loss values for each model with increasing epoch values in apple leaf disease detection. In general, the loss functions of each model decreased with increasing epochs and eventually stabilized. The SSD model had the fastest convergence of the training loss curve, but also had the largest loss value after stabilization, which reached full convergence after 10 epochs. The loss functions of the other six models gradually stabilized after 60 epochs of training. Among them, MGA-YOLOV5 had the second-highest training loss value after the training loss function gradually stabilized. The loss functions of YOLOV5-CBAM-C3TR, BTC-YOLOV5, YOLOV3, and YOLOV4 were very similar, with slightly higher stabilized loss values than those of YOLOV5. YOLOV5 has the lowest training loss value of all the models.

**Figure 4B** shows a plot of the validation loss function with increasing epoch values. As with the training loss function curve, the SSD model still had the fastest convergence rate and stabilized after 10 epochs, while the validation loss value was the highest. The other six target detection models all stabilized around the 30th epoch. Specifically, YOLOV5 and MGA-YOLOV5 had the similar loss function curves after stabilization, with the second highest loss function value. The YOLOV3, YOLOV4, BTC-YOLOV5, and YOLOV5-CBAM-C3TR had also the similar loss curves after stabilization, with slightly higher stabilized loss values than that of YOLOV5, which had the lowest training loss value among all models.

### 3.3 Comparative analysis with different detection models

The objective of this study is to propose a target detection model capable of accurately identifying and locating apple leaf diseases, which can assist the disease precision spraying device for automatic spraying. To verify the effectiveness of YOLOV5-CBAM-C3TR in detecting apple leaf diseases, it was compared with SSD, YOLOV3, YOLOV4, YOLOV5, MGA-YOLOV5 and BTC-YOLOV5 models on the same dataset. The results in **Table 3** showed that the mAP 0.5and mAP@[0.5:0.95] of YOLOV4 were the lowest with 52.4% and 18.6% respectively. While the mAP 0.5and mAP@[0.5:0.95] of YOLOV5-CBAM-C3TR were the highest with 73.4% and 40.9% respectively. The precision of SSD was up to 96.8% and the recall was only 36.9%, indicating that SSD was accurate in detecting apple leaf diseases, but there were omissions in disease identification. Similarly, YOLOV4 had large variations in precision and recall, resulting in a poor mAP 0.5 In contrast, YOLOV3, YOLOV5, MGA-YOLOV5, and BTC-YOLOV5 can balance the precision and recall metrics better, with mAP 0.5of 65.1%, 67.8%, 69%, and 72%, respectively. Overall, compared with YOLOV5, the optimized YOLOV5-CBAM-C3TR showed a significant improvement in detection precision, with an 8.25% improvement in mAP@0.5 and a 17.2% improvement in mAP@[0.5:0.95]. In addition, the experimental results also confirms that the optimized YOLOV5-CBAM-C3TR has a high detection accuracy, which is sufficient for practical needs.

### 3.4 Performance of the improved model in apple leaf disease detection

**Figure 5:**
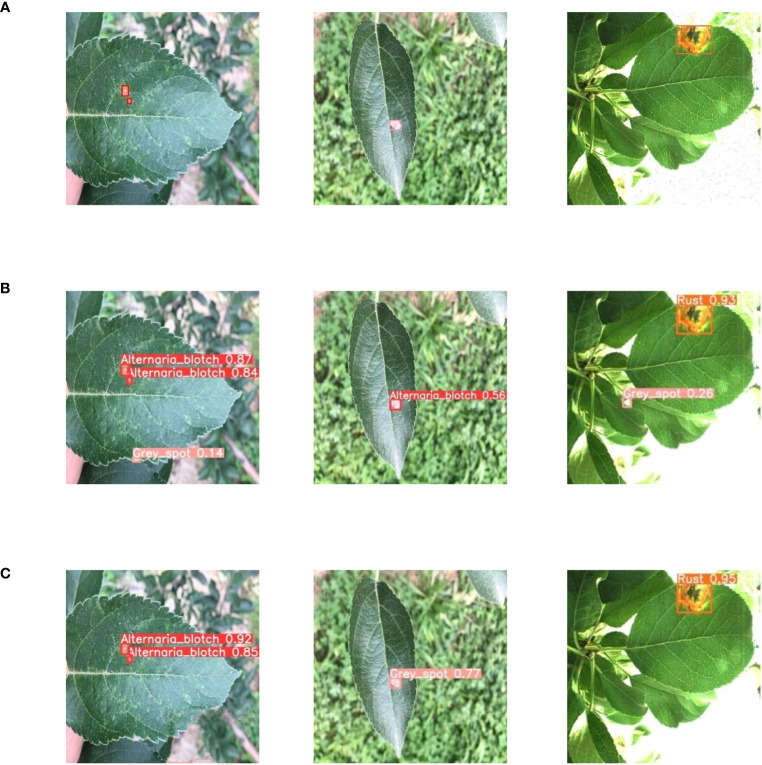
**(A)** Original image, **(B)** YOLOV5 detection results, **(C)** YOLOV5-CBAM-C3TR detection results. Different colored bounding boxes are used in the images to distinguish the types of apple leaf diseases, with Alternaria blotch in red, Grey spot in pink and Rust in orange. The names of apple leaf diseases from the first to the third column in the image are: Alternaria blotch, Grey spot, and Rust.

**Figure 6:**
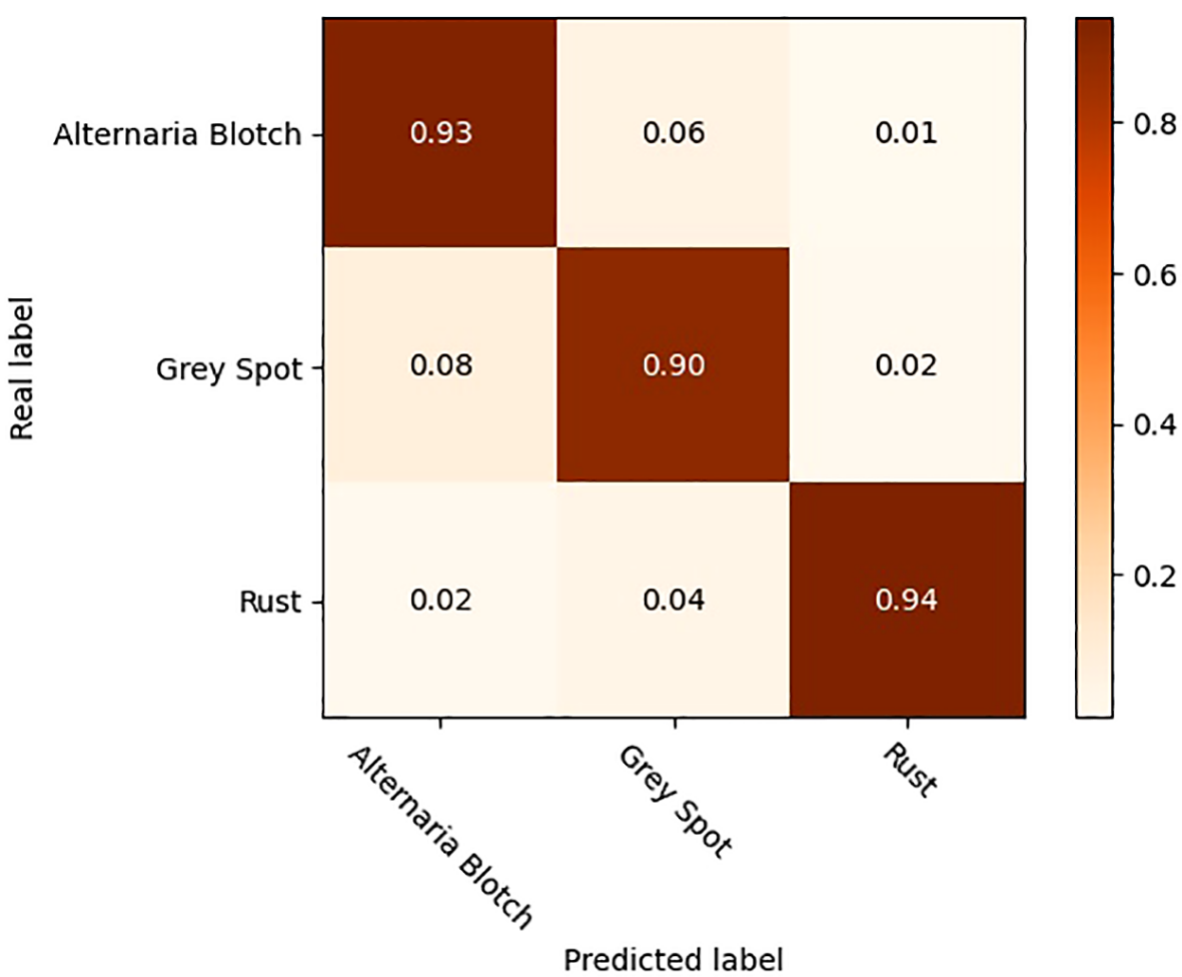
Confusion matrix for the detection of three apple leaf diseases.

To further validate the effectiveness of the improved model, the original YOLOV5 model and the optimized YOLOV5-CBAM-C3TR model were selected for the comparison of detection results in real environments. A total of 208 sample images with natural environment backgrounds were selected in the test set to examine the detection effect of YOLOV5-CBAM-C3TR in real scenes. **Table 6** shows that YOLOV5-CBAM-C3TR improves the correct recognition rate of the three apple leaf diseases compared to YOLOV5, with a significant increase of 18.9% in the average accuracy. **Figure 5** shows a comparison of typical detection results for the three apple leaf diseases. The results show that the YOLOV5 algorithm has errors in detecting the three apple leaf diseases in a natural environment with a complex background, and the main reason for the unsatisfactory detection results is its inaccurate feature extraction of the diseases. As can be seen in **Figure 6**, YOLOV5-CBAM-C3TR is able to extract the features of various diseases better, but there is still a risk of misjudging Alternaria blotch and Grey spot, which are two similar diseases. The possible reason for this is that these two diseases are very similar after data enhancement in the simulated natural environment. In this study, the CBAM module and the C3TR module were added to YOLOV5, and the two modules work together to enable YOLOV5 to better extract disease features.

**Table 6:** Test results of improved models in detecting apple leaf diseases.

Model	Number of Alternaria Blotch Samples	Number of Correctly Detected	Number of Grey Spot Samples	Number of Correctly Detected	Number of Rust Samples	Number of Correctly Detected	Average Accuracy (%)
YOLOV5	87	52	67	54	54	50	77.7
YOLOV5-CBAM-C3TR	87	81	67	60	54	51	92.4

## 4 Discussion

In this study, attention mechanism and module with the transformer encoder were added to optimize YOLOV5, and finally proposed YOLOV5-CBAM-C3TR to accurately classify three common diseases of apple leaves. Comparing with the target detection algorithms such as SSD, YOLOv3, YOLOv4, and YOLOv5, YOLOV5-CBAM-C3TR had the highest mAP@0.5 and mAP@[0.5:0.95], which reached 73.4% and 40.9%, respectively. An average accuracy of 92.4% was achieved on a randomly selected sample of 208 images containing the three apple leaf diseases. Empirical results showed that adding CBAM, SE, ECA, and C3TR individually or in combination can significantly improve the detection accuracy of YOLOV5. In contrast, combining each attention mechanism with the C3TR module has a higher detection accuracy than adding each module separately. Among them, the combination of CBAM and C3TR provided the most significant performance enhancement for YOLOv5. Different from SE or ECA modules, CBAM module pays attention to both channel information and spatial information, and can better cooperate with C3TR for global information extraction. Certainly, Attention mechanisms have been shown to be effective in many tasks (Xue et al., 2021; Wang et al., 2022; Zhao et al., 2022). However, the task requirements in different scenarios should be carefully considered when choosing the appropriate attention module, which suggests that the selection of modules requires extensive experimentation. Although adding modules can improve the detection accuracy of target objects, it also increases the number of parameters of the model, which is not conducive to the actual deployment of the model. The addition of CBAM, ECA, SE, and C3TR in this study increased the number of parameters in YOLOV5. Future research will consider methods to reduce the number of parameters while maintaining model detection accuracy, such as pruning (Liang et al., 2022) and distillation, in order to achieve a good balance between model detection accuracy and the number of parameters.

Timely detection and control of apple leaf diseases is extremely important. Since different apple leaf diseases may have similar characteristics, even the human eye cannot distinguish them accurately after exposure and other treatments that simulate the natural environment. The experimental data in **Table 6** showed that YOLOV5-CBAM-C3TR improved the two types of diseases including Alternaria blotch and Grey spot, by 33.33% and 8.95%, respectively. The average accuracy achieved 92.1% for the three types of diseases. The experimental data affirmed the ability of the optimized model to accurately identify similar diseases. However, factors such as the number of disease types in the data set, the complexity of the environment of the objects to be detected in the image, and the difference in categories of the objects to be detected will affect the accuracy of the network model detection to some extent. Therefore, the selection of detection accuracy to evaluate the model performance should be combined with specific application scenarios. For example, Khan et al. (2022) developed an automated apple leaf disease detection system based on deep learning. The experimental results show that on a dataset containing more than 9000 images, Faster-RCNN can reach 42.01% mAP at 6FPS, showing a good detection accuracy for 9 common apple leaf diseases. The model is tested on data set images that are less disturbed by the real background environment, and its robustness is low in the real environment. The experimental results also indicate that the model is not effective in detecting diseased leaves. When using a target detection model to detect apple leaf diseases, Zhang et al. (2023) found that the MFaster R-CNN model could achieve 97.23% mAP for eight kinds of corn leaf diseases, but only 80.69% mAP on a self-built data set of apple leaf diseases. The above examples show that the accuracy evaluation of the model should be combined with the specific application scenarios of the model. In different tasks and different data sets, the model will show different detection performance. Only 3 kinds of apple leaf diseases were considered in this experiment, while there are more than 200 kinds of apple leaf diseases. Therefore, although YOLOV5-CBAM-C3TR can accurately identify apple leaf diseases similar to those in the classification data set, it may not be universally applicable to other similar diseases. It is necessary to expand the data set of apple leaf disease and collect more comprehensive types of leaf disease for research. In addition, the model proposed in this study needs to be compared with more advanced object detection algorithms such as YOLOV7 and YOLOV8. These questions will be further explored in the future to improve the accuracy of the model’s detection of different apple leaf diseases so that each class of similar diseases can be accurately classified.

## 5 Conclusions

YOLOV5-CBAM-C3TR algorithm was proposed to improve the accuracy of detection of three apple leaf diseases including Alternaria blotch, Grey spot, and Rust. The model was obtained by optimizing YOLOV5 with the addition of an attention mechanism and a module with a transformer encoder. Compared with different target detection models, the optimized YOLOV5-CBAM-C3TR algorithm achieved the highest detection accuracy than other models, with mAP@0.5, mAP@[0.5:0.95], precision, recall of 73.4%, 40.9%, 70.9%, 69.5%, respectively. In randomly selected apple leaf disease samples, the average accuracy based on the YOLOV5-CBAM-C3TR model can reach 92.4%, which was 18.9% higher than that of the original YOLOV5. Moreover, the YOLOV5-CBAM-C3TR model also showed a strong ability to identify similar diseases, and could accurately identify Alternaria blotch and grey spot, which are almost indistinguishable from the naked eye. In the future, YOLOV5-CBAM-C3TR can also be extended to detect similar diseases in other crops.

## Data availability statement

The original contributions presented in the study are included in the article/supplementary material. Further inquiries can be directed to the corresponding author.

## Author contributions

ML: Data curation, Formal analysis, Investigation, Methodology, Software, Validation, Visualization, Writing – original draft. W-HS: Conceptualization, Funding acquisition, Methodology, Project administration, Resources, Supervision, Writing – review & editing.
